# Effect of Low-Intensity Aerobic Exercise on Insulin-Like Growth Factor-I and Insulin-Like Growth Factor-Binding Proteins in Healthy Men

**DOI:** 10.1155/2010/452820

**Published:** 2010-09-22

**Authors:** Yuichiro Nishida, Takeshi Matsubara, Takuro Tobina, Munehiro Shindo, Kumpei Tokuyama, Keitaro Tanaka, Hiroaki Tanaka

**Affiliations:** ^1^Laboratory of Exercise Physiology, Faculty of Health and Sports Science, Fukuoka University, Fukuoka 814-0180, Japan; ^2^Department of Preventive Medicine, Faculty of Medicine, Saga University, 5-1-1 Nabeshima, Saga 849-8501, Japan; ^3^Laboratory of Physical Science Inc., Fukuoka 810-0001, Japan; ^4^Laboratory of Biochemistry of Exercise and Nutrition, Institute of Health and Sport Sciences, University of Tsukuba, Ibaraki 305-8574, Japan

## Abstract

Increased concentrations of circulating insulin-like growth factor-I (IGF-I) or IGF-I relative to IGF-binding proteins (IGFBPs) are associated with increased risk of developing several forms of cancer. Conversely, exercise is linked with reduced risk. This study aims to investigate the effect of a low-intensity exercise program on circulating levels of IGF-I, IGFBP-1, and IGFBP-3, in previously sedentary males. Fourteen healthy men participated in cycle ergometer training at lactate threshold intensity for 60 min/day, 5 days/week for 6 weeks. After aerobic training, insulin sensitivity improved by 20%, while fasting insulin levels decreased by 13%. Simultaneously, low-intensity aerobic training decreased the circulating levels of IGF-I by 9%, while IGFBP-1 levels increased by 16%. An interesting finding was that higher pretraining level of IGF-I was associated with greater decline in IGF-I with training. Insulin-sensitizing low-intensity aerobic exercise is thus considered to be an effective method for downregulating IGF-I and upregulating IGFBP-1 levels.

## 1. Introduction

Strong epidemiological evidence indicates that a sedentary lifestyle leads to an increased risk of developing certain cancers, including colon, prostate, breast, and endometrial cancers [1–3]. By contrast, physical activity has been shown to have a protective effect against their development. IGF-I is a potent mitogen that promotes cellular proliferation and prevents apoptosis in normal and cancer cells [4–6]. IGF-I axis hormones are important factors implicated in the beneficial influence of exercise. Epidemiological studies have shown that increased levels of circulating IGF-I or increased IGF-I relative to IGFBP-3 are associated with a higher risk of developing several forms of cancer, including prostate and breast cancers [7–12].

The combination of a low-fat diet and exercise program is known to reduce IGF levels. Barnard et al. [13, 14] showed a decrease in the serum concentration of IGF-I and an increase in IGFBP-1 with the implementation of a low-fat diet and exercise program. Furthermore, they reported that serum from men undergoing this form of intervention showed reduced cancer cell growth and increased apoptosis in LNCaP prostate cancer cells *in vitro* [13, 15]. In conjunction with the changes in serum IGF-I and IGFBP-1, reductions have also been shown in insulin and free testosterone, along with increases in sex hormone-binding globulin [15, 16]. The changes in IGF-I and IGFBP-1 appear to be particularly important. When IGFBP-1 was added to preintervention serum, LNCaP cancer cells underwent apoptosis; whereas, when IGF-I was added to postintervention serum, the reduction in growth was eliminated [17]. Thus, the down-regulation of IGF-I and up-regulation of IGFBP-1, as a result of the diet modification and exercise intervention program, may have protective effects against the development of cancerous cells. However, the training intensity and/or duration of exercise required to induce such favorable alterations has not yet been fully elucidated.

A number of intervention studies have shown increased [18–23], decreased [24, 25], or unaltered [26, 27] levels of IGF-I after endurance or resistance training. Similarly, many cross-sectional observational studies have examined the association of physical activity with IGF-I levels in the general population; however, their findings have been inconsistent [28–32]. Additionally, some reports have shown that circulating levels of IGFBP-1 and IGFBP-3 are also modulated with exercise [23, 33, 34], whereas other groups saw no effects in their levels with moderate-intensity aerobic exercise or strength training [35, 36]. The different responses of IGF axis hormones to the intensity and/or duration of various types of exercise could be the reason why exercise is not necessarily protective against all forms of cancer. Furthermore, it also has been reported that very heavy exercisers demonstrate high mortality ratios for lung, colorectal, and pancreatic cancers [37].

Whether exercise induces desirable (i.e., downregulation of IGF-I and upregulation of IGFBP-1) or undesirable (i.e., up-regulation of IGF-I and down-regulation of IGFBP-1) effects on cancer prevention is highly dependent on training intensity. As high-intensity exercise produces an acute increase in circulating IGF-I levels [38, 39], the exercise intervention that induces a reduction in IGF-I levels may be in the low or moderate categories. To the best of our knowledge, only one cross-sectional study has shown a reduction in serum IGF-I levels and increase in IGFBP-1 levels in participants of an exercise program, without emphasis on diet modification, in comparison to a control group [16]. Despite the fact that physical exercise is considered a strong intervention for cancer prevention [1], there are few studies that specifically assess the effects of exercise on specific biomarkers of cancer [40]. In addition, it has been described in a previous review that persons at varying risks of developing cancer should be examined in exercise intervention studies, as the magnitude of protection caused by exercise may differ in high-versus low-risk individuals [40]. Furthermore, no previous intervention study has been conducted to examine whether an exercise regimen alone can elicit favorable changes in both IGF-I and IGFBP-1 levels for cancer prevention in healthy men. Additionally, insulin is another factor which has the potential to influence the levels of IGF-I and IGFBP-1 after exercise training. Hyperinsulinemia stimulates liver production of IGF-I and suppresses IGFBP-1 production [4, 41]. It is also known that insulin itself promotes cellular growth in normal as well as malignant tissues [42, 43]. In addition, leptin may also regulate changes in IGF-I following exercise intervention [44].

The lactate threshold (LT) represents the oxygen uptake (VO_2_) or work rate above which there is a systemic rise in blood lactate levels during incremental exercise [45]. The LT level corresponds to approximately 50%  VO_2max_, and exercise at LT can be performed easily and safely even in the elderly and individuals with metabolic syndrome [46, 47]. In a previous study by our group, low-intensity aerobic training at LT level caused an increase in insulin sensitivity (S_I_), with a concomitant decrease in the basal level of insulin [48]. In this study we used this exercise training program as it was designed to have an insulin-sensitizing effect. We hypothesized that LT-level aerobic training, which does not stimulate IGF-I production and reduces basal insulin levels, would result in simultaneous alterations in both the down-regulation of IGF-I and up-regulation of IGFBP-1. Thus, we investigated the effect of mild aerobic exercise training at LT level on the circulating levels of IGF-I, IGFBP-1, and IGFBP-3 to determine an optimal exercise intervention that can induce favorable changes in IGF-I and IGFBP-1 levels for cancer prevention in healthy men.

## 2. Methods

### 2.1. Subjects

Fourteen healthy men (22.6 ± 0.5 years) who had not undergone any regular exercise for at least 2 years were examined. All subjects were nonsmokers, had no evidence of chronic disease, such as diabetes, hypertension or cancer, and were not taking any medication. All subjects were also asked to maintain their normal dietary habits and not to engage in any strenuous physical activity. Before beginning the study, the nature, purpose, and risks of the study were explained to all subjects, and informed written consent was obtained. The protocol was approved by the local ethical committee of Fukuoka University.

### 2.2. Body Composition, Physical Fitness, and Exercise Training

Each subject's body fat percentage was measured by hydrostatic weighing before commencing training and 2 days after the last training session. This was estimated based on the hydrostatic density with a correction for the residual lung volume. To measure physical fitness, a graded exercise test was performed on a mechanically braked ergometer (Electric Bicycle Ergometer, Lode's Instrumenten B. V., Groningen, Holland) before commencing the training program and 2 days after the final training session. The work rate was initially set at 10 watts and increased every 4 seconds by 1 watt, until physical exhaustion. VO_2_ was measured from the mixed expired gas collected in neoprene bags. The volume of the expired gas was quantified with a twin-drum-type respirometer (Fukuda Irika CR-20, Tokyo, Japan), and both the O_2_ and CO_2_ fractions were analyzed by a mass spectrometer (ARCO-1000, ARCO System Inc., Chiba, Japan). Blood samples were obtained from the earlobe every 30 seconds to measure blood lactate levels. The blood lactate concentration was plotted against the exercise workload for each subject, and the workload at the first breaking of lactate was used to calculate the exercise training intensity for each subject. The LT was determined for each subject based on a visual inspection, according to the estimations of three experts, who were blinded to the purpose of our study, and the average was used to establish the exercise intensity for training. Cycle ergometer aerobic training at the LT level was performed at our laboratory for 60 minutes per day, five times a week for 6 weeks.

### 2.3. Blood Sampling and Analysis

Blood samples were obtained from an antecubital vein each morning between 0700 and 0900 h, following overnight fasting, prior to training, and 16–72 hours after the final training session. Plasma glucose levels were measured spectrophotometrically using glucose oxidase (Glucose B-test; Wako Pure Chemical, Osaka, Japan); serum insulin (Phadeseph insulin radioimmunoassay kit, Shionogi, Osaka, Japan) and leptin (Human leptin kit, LINCO Research, Missouri, USA) concentrations were measured by radioimmunoassay; and serum IGF-I (Somatomedin-C*·*II [Chiron] measurement kit, Chiron Inc., Tokyo, Japan), IGFBP-1 (DSL-7800, Diagnostic Systems Laboratories, Inc., Texas, USA), and IGFBP-3 (Ab Tube IGF-BP-3 Eiken, Diagnostic Systems Laboratories, Inc., Texas, USA) were measured by immunoradiometric assay. The intra- and interassay coefficient variations were 3.9% and 2.8% for IGF-I, 4.6% and 6.0% for IGFBP-1, and 7.2% and 10.5% for IGFBP-3, respectively. Pre- and posttraining samples were measured simultaneously. Insulin resistance index (IRI) was calculated using Matthews' formula [49].

### 2.4. Intravenous Glucose Tolerance Test (IVGTT) and Minimal Model Data Analysis

IVGTTs were performed before commencing the training program and 16 hours after the last training session. Following overnight fasting, subjects were allowed to rest while lying down for at least 30 minutes prior to blood sampling. Baseline samples for glucose and insulin were obtained, followed by glucose administration via the contralateral antecubital vein (300 mg/kg body weight) within 2 minutes. Subsequent samples were obtained at frequent intervals until 180 min, as previously described [50]. Insulin (Humalin; Shionogi, Osaka, Japan) was infused (20 mU/kg) via an antecubital vein between the periods of 20–25 minutes post glucose administration. On the day before undergoing IVGTT, all subjects were provided with an evening meal consisting of ≥140 g carbohydrate, ≥30 g fat, and ≥33 g protein. S_I_ was estimated using a minimal model approach, as previously described [50]. The S_I_ index represents the net increase in glucose disappearance rate, which also depends on the rise in insulin above basal levels. The minimal model program was written using Pascal programming (Borland International, CA) on a Macintosh IIcx (Apple Computer, CA).

### 2.5. Statistics

All values are shown as the means ± standard error (SE). Statistical analyses were performed using Wilcoxon's signed-rank test. Pearson's correlation coefficient was used for the analysis of the correlation between the changes of variables after the exercise training. A *P* value of less than  .05 was considered to be statistically significant.

## 3. Results

Weight and body fat percentage did not change after the training period (Table 1). By contrast, mild training significantly increased indices of aerobic fitness. Both basal glucose and insulin levels significantly reduced after training (Table 2). Although IRI, which was calculated using Matthews' formula, increased only slightly, the increase was significant. Circulating leptin levels were not influenced by training. LT exercise was found to significantly decrease the circulating levels of IGF-I, whereas IGFBP-1 was significantly increased. By contrast, IGFBP-3 levels were not influenced by training.

**Figure 1:**
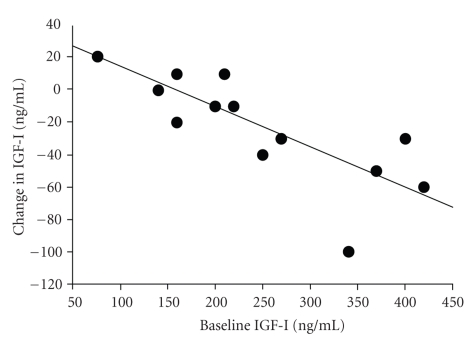
Relationship between pre-training IGF-I level and individual changes in IGF-I after training.

**Figure 2:**
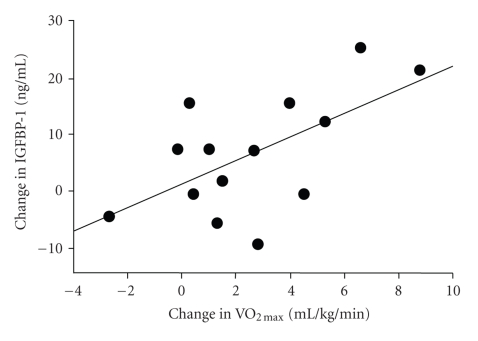
Changes in VO_2max _and circulating IGFBP-1 levels after low-intensity exercise.

There was no significant relationship between the baseline level of IGF-I and baseline measures of VO_2max _ (*r* = 0.15) or LT-VO_2_ (*r* = 0.20). There were also no significant correlations between the change in circulating IGF-I levels and the changes in aerobic fitness (VO_2max_, *r* = − 0.10), basal insulin (*r* = 0.08), IRI (*r* = 0.12), and S_I_ (*r* = 0.41) after exercise intervention. Similarly, relationships between the change in IGFBP-1 and changes in basal insulin (*r* = −0.33) and S_I_ (*r* = 0.27) were not statistically significant. Furthermore, the change in leptin levels after exercise was not significantly correlated with the changes in IGF-I (*r* = −0.22) or IGFBP-1 (*r* = −0.45). Conversely, there was a negative correlation between pre-training IGF-I levels and individual changes in IGF-I after training (*r* = −0.77; *P* < .01) (Figure 1). Changes in VO_2max _ showed a positive correlation with changes in IGFBP-1 (r = 0.61; *P* < .05) (Figure 2)_._

## 4. Discussion

The main findings of this study were as follows: (1) short-term cycle ergometer aerobic training at LT level decreases circulating IGF-I concentrations and increases IGFBP-1 levels, without changing body weight, in previously sedentary men; (2) there is an inverse relationship between pre-training IGF-I levels and individual changes in IGF-I after training, suggesting that individuals with a higher pre-training IGF-I level will have a more substantial decrease in IGF-I following exercise intervention; and (3) increases in aerobic fitness (VO_2max _) positively correlates with changes in IGFBP-1 level after training. It is known that alterations in the IGF axis, including a reduction in IGF-I and an increase in IGFBP-1, by lifestyle modification are associated with an *in vitro* reduction in prostate cancer cell (LNCaP) growth and increased apoptosis [13, 16]. Thus, the current insulin-sensitizing exercise program is a safe and easily-performed exercise program that simultaneously induces a down-regulation of IGF-I and up-regulation of IGFBP-1.

The physiological effects of decreased IGF-I and increased IGFBP-1 levels after mild aerobic training have not been clarified by this study. However, since IGF-I infusion causes hypoglycemia, primarly by stimulating peripheral glucose uptake [51], and IGFBPs buffer the acute hypoglycemic effect of IGF-I [52], we thus speculate that the alterations in IGF-I and IGFBP-1 levels may be an adaptive response to prevent hypoglycemia following insulin-sensitizing training. In a previous study of exercise-induced energy deficit, leptin administration significantly increased circulating levels of IGF-I in healthy men and women [44]. In this study, leptin levels were unchanged after the training program, and the individual changes in the levels of IGF-I were not significantly correlated with the changes in leptin concentration. Thus, leptin may not play a role in the alteration of IGF-I after mild exercise in healthy men.

The principal factor enhancing the production of IGF-I in the liver is growth hormone, which stimulates IGF-I synthesis and is further enhanced by insulin [41, 52]. As fasting insulin levels are slightly but significantly decreased after exercise intervention, it could potentially contribute to the reduction in IGF-I levels. Some [19, 22, 39], but not all [53–55], previous studies have reported increases in IGF-I after acute exercise; however, these increases are transient and typically return to baseline levels within 10–15 min after exercise [56]. Increases in IGF-I after acute exercise are considered to be unrelated to exercise-induced increases in growth hormone [56]. Moreover, increases in IGF-I after acute exercise were observed in growth hormone-deficient subjects [19]. We previously showed that acute bout of exercise at LT transiently increases growth hormone level [57]. However, it is likely that resting levels of growth hormone are not influenced by low-intensity training [58].

It is worthwhile noting that this study examines changes in systemic IGF-I levels and that changes in local production (i.e., paracrine/autocrine effects) are not assessed. Thus, this study could not capture the potential effect of exercise on IGF changes at the tissue level. A single bout of acute resistance exercise upregulates local (i.e., skeletal muscle) IGF-I [59–61]. To the best of our knowledge, whether local IGF-I is upregulated by low-intensity aerobic exercise remains unknown. A recent review suggested that local changes in IGF-I are independent of changes in circulating IGF-I, indicating that serum IGF-I is not necessarily a reflection of local concentrations [56]. Thus, in this study, the reduced levels of systemic IGF-I post-exercise intervention may not have been influenced by local IGF-I.

It has been considered that reduced insulin levels after exercise may contribute to the up-regulation of IGFBP-1 [14]. Furthermore, exercise-induced changes in basal insulin levels did not significantly correlate with changes in IGFBP-1; however, this may be due to the low number of subjects. Conversely, the present results show a significant relationship between changes in VO_2max _ and circulating IGFBP-1 levels. Additionally, it seems noteworthy that correlations between the change in IGFBP-1 and changes in basal insulin (*r* = −0.33) and S_I_ (*r* = 0.27) were moderate and in the expected direction, although not statistically significant. The lack of statistical significance is most likely due to the low sample size. Although we are unable to explain the underlying mechanisms, the enhancement of aerobic fitness might be important in the up-regulation of IGFBP-1 with low-intensity training. It was shown by Hellenius et al. [62] that low-intensity aerobic exercise (2 to 3 times/week at an intensity of 60–80% maximal heart rate for 30–45 min) for 6 months improved S_I_ and increased IGFBP-1 in healthy middle-aged men. Although that study did not assess the level of aerobic fitness (VO_2max _), fasting insulin levels decreased in the exercise group by 14%, with a 15% increase in IGFBP-1 after training for 6 months. In this study a similar decrease in fasting insulin (13%) caused a similar increase in IGFBP-1 (16%) after exercise intervention. This indicates that increases in IGFBP-1 are regulated by changes in insulin induced by exercise training.

A previous study showed that the changes in IGF-I and IGFBP-1 levels, induced by a combined low-fat diet and exercise program, were accompanied by a reduction in body weight [16]. Furthermore, in this cross-sectional study, participants in the exercise program with a lower IGF-I and higher IGFBP-1 had a much lower BMI, compared with the control group [16]. By contrast, another report on the effects of aerobic exercise combined with a low-fat, high-complex carbohydrate diet showed reduced serum insulin and positive control over aspects of metabolic syndrome, including hypertension and hypertriglyceridemia, over a period of only 3 weeks, even though the subjects remained overweight or obese [63]. Furthermore, using regression analyses, Nemet et al. [64] examined IGF-I and body mass changes associated with 7-day strenuous exercise and found a decrease in IGF-I levels, even in weight-stable subjects. Similarly, this study showed that alterations of circulating IGF-I and IGFBP-1, basal insulin, and S_I_ after LT-level training were observed without changes in body weight and body fat percentage. Thus, in conjunction with previous results, this suggests the importance of exercise alone, rather than changes in body composition, on the regulation of IGF-I and IGFBP-1.

There were some limitations of this study that should also be described. The short period (6 weeks) of training intervention was a limiting factor in this study. Further studies are needed to examine whether longer periods of exercise intervention induce sustained and greater impacts on the changes in insulin sensitivity, fasting insulin, and IGF-I and IGFBP-1 levels. Information on dietary intake before and during the intervention was not recorded, despite the fact that diet is an important modifier of IGF levels. Because of this limitation, we are unable to perform a cross-sectional analysis of the relationship between dietary components and IGF levels. It is already known that IGF-I declines during energy and protein restriction [41]. Since body weight did not decrease in this study, dietary intake does not need to be reduced during the exercise intervention. Thus, reduced IGF levels after the current aerobic training might not be affected by decreased dietary intake. Another limitation is that this study has no control group. The potential problems with the absence of a control group are the unwitting incorporation of a sampling bias. For example, the possibility that an inverse relationship between the initial (baseline) IGF-I levels and the change in IGF-I levels after training could be due to a convergence towards the mean effect cannot be fully excluded. However, previous reports have shown that a single IGF-I measurement is generally representative of the levels over a period of time [7] and IGF-I levels appear to have no detectable diurnal or circadian variation [29, 65].

It is also worth mentioning that, although a lower level of IGF-I is associated with a lower cancer risk in prospective healthy population, several studies have shown that lower IGF-I is associated with an increased risk of cardiovascular disease, type II diabetes, obesity [56, 66], osteoporosis, and cognitive decline [56]. There are also reports of conflicting data showing that subjects with obesity or type II diabetes have normal levels of total IGF-I [67, 68] and no significant correlation between IGF-I and bone mineral density in women [69, 70]. Nindl and Pierce have described in their recent review that the fact that there are studies claiming that both increases and decreases in IGF-I concentrations have beneficial effects on health presents a contradictory situation. Furthermore, they also stated that, even though local IGF-I is consistently up-regulated with both acute and chronic exercises, circulating IGF-I may actually decrease [56]. However, despite lowering IGF-I, the current exercise program would be expected to have beneficial effects on all of these various conditions.

In this study, subjects were healthy men with a relatively low risk of developing cancer. Although the changes in IGF-I were most dramatic in men with high baseline levels, it is not clear whether this will apply to populations with different baseline levels. Serum levels of IGF-I are inversely associated with age [29, 31], and IGF-I levels are higher in women than in men in Western population [29]. Conversely, in the Japanese population, the circulating IGF-I level was found to be higher in men than in women [32]. Further work is needed to extend the present outcome to a wider population by examining whether the same exercise regimen at the LT can induce such favorable changes in individuals who have elevated IGF-I levels and are at increased risk of developing cancer.

In conclusion, we found that short-term aerobic exercisetraining at LT levels decreased circulating IGF-I and increased IGFBP-1 levels, without changing body composition, in previously sedentary men. These results are consistent with those of a previous rodent study demonstrating the beneficial effects of low-level exercise on cardiovascular, as well as cancer risk factors [71]. It has been described that physical exercise deserves particular attention in the prevention of neoplasia, especially as it also exerts consistent beneficial effects on other major chronic diseases prevalent in the Western world, such as atherosclerosis and type 2 diabetes [1]. The current low-intensity aerobic training regimen is thus considered to be an effective approach in healthy men for down-regulating IGF-I and up-regulating IGFBP-1 levels.

## Figures and Tables

**Table 1:** Characteristics of the subjects.

	Before training	After training
Age (year)	22.6 ± 0.5	
Height (m)	1.71 ± 0.01	
Weight (kg)	63.5 ± 1.8	62.9 ± 1.8
BMI (kg/m^2^)	21.7 ± 0.6	21.4 ± 0.6
Percent fat (%)	13.6 ± 1.1	12.9 ± 1.1
Fat mass (kg)	8.7 ± 0.9	8.2 ± 0.8
LBM (kg)	54.8 ± 1.4	54.7 ± 1.5
VO_2max _ (mL/kg/min)	42.7 ± 1.1	45.4 ± 1.0*
LT-VO_2_ (mL/kg/min)	18.1 ± 0.9	23.5 ± 0.7*

Values are the means ± SE. BMI, body mass index; LBM, lean body mass; LT-VO_2_, VO_2_  at lactate threshold. *P < .05 versus before training.

**Table 2:** Metabolic parameters: IGF-I, IGFBP-1, and, IGFB-3 before and after mild training.

	Before training	After training
*n*	14	14

Basal glucose (mg/dL)	93.0 ± 1.0	90.3 ± 1.3*
Basal insulin (*μ*U/mL)	4.7 ± 0.2	4.1 ± 0.2*
IRI (*μ*U*·*mL^−1^ *·*mg*·*dL^−1^)	1.08 ± 0.06	0.91 ± 0.05*
S_I_ (×10^−5^ *·*min*·* *μ*U*·*mL^−1^)	16.2 ± 1.5	19.5 ± 1.4*
Leptin (ng/mL)	1.9 ± 0.3	1.7 ± 0.1
IGF-I (ng/mL)	245 ± 28	223 ± 22*
IGFBP-1 (ng/mL)	39.4 ± 2.8	45.7 ± 3.8*
IGFBP-3 (ng/mL)	2665 ± 129	2583 ± 110

Values are the means ± SE. IRI, insulin resistance index;  S_I_, insulin sensitivity; IGF-I, insulin-like growth factor-I; IGFBP, IGF-binding protein. *P < .05 versus before training.
